# Giant exchange interaction in mixed lanthanides

**DOI:** 10.1038/srep24046

**Published:** 2016-04-18

**Authors:** Veacheslav Vieru, Naoya Iwahara, Liviu Ungur, Liviu F. Chibotaru

**Affiliations:** 1 Theory of Nanomaterials Group, Katholieke Universiteit Leuven, Celestijnenlaan 200F, B-3001 Leuven, Belgium

## Abstract

Combining strong magnetic anisotropy with strong exchange interaction is a long standing goal in the design of quantum magnets. The lanthanide complexes, while exhibiting a very strong ionic anisotropy, usually display a weak exchange coupling, amounting to only a few wavenumbers. Recently, an isostructural series of mixed 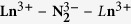 (Ln = Gd, Tb, Dy, Ho, Er) have been reported, in which the exchange splitting is estimated to reach hundreds wavenumbers. The microscopic mechanism governing the unusual exchange interaction in these compounds is revealed here by combining detailed modeling with density-functional theory and *ab initio* calculations. We find it to be basically kinetic and highly complex, involving non-negligible contributions up to seventh power of total angular momentum of each lanthanide site. The performed analysis also elucidates the origin of magnetization blocking in these compounds. Contrary to general expectations the latter is not always favored by strong exchange interaction.

The effects of strong magnetic anisotropy, traditionally investigated in magnetic insulators, especially, in *f*-electron systems^1^,^2^,^3^, recently attracted renewed interest in connection with molecular magnetic materials^4^. The investigation of molecular nanomagnets gave birth to new objects such as single-molecule magnets (SMMs)^5^,^6^ and single-chain magnets^7^, and initiated studies in the domain of molecular spintronics^8^,^9^ and quantum computation^10^,^11^,^12^. Among them, in the last years the accent moved towards lanthanide complexes which have already demonstrated several exciting properties^13^,^14^,^15^,^16^,^17^,^18^.

The key feature of lanthanide ions in materials is their strong magnetic anisotropy caused by strong spin-orbit coupling effects^19^, which often leads to highly axial ground and low-lying excited doublet states even in the lack of axial symmetry^20^. Due to small radius of electronic *f*-shells, the exchange interaction in lanthanide complexes is much weaker than the crystal-field splitting on lanthanide ions^21^. As a result, only individual doublet states on lanthanide sites, described by pseudospins 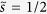, participate in the magnetic interaction. The latter is described by a Hamiltonian bilinear in pseudospins (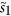 and 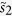) in the case of two interacting lanthanide ions, or a pseudospin 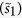 and a true spin (*S*_2_) in the case of a lanthanide ion interacting with a transition metal or a radical when the spin-orbit coupling in the second site is negligible. For strongly axial doublet states on the lanthanide sites (Ln) these Hamiltonians basically become of Ising type^22^:


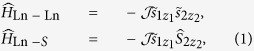


either collinear (*z*_1_ ‖ *z*_2_) or non-collinear 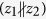 depending on geometry^21^ and details of interaction^23^. The exchange parameter is contributed by magnetic dipolar and exchange interaction between the sites, 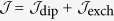, the former being usually stronger in net lanthanide complexes^21^.

This paradigm was recently challenged by a series of 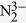-radical bridged dilanthanide complexes [K(18 − crown − 6)]{[(Me_3_Si)_2_N] (THF)Ln}_2_ (*μ* − *η*^2^:*η*^2^ − N_2_) (Ln = Gd (**1**), Tb (**2**), Dy (**3**), Ho (**4**), Er (**5**), THF = tetrahydrofuran), shown in Fig. 1a^14^,^24^. In some of these compounds the exchange interaction was found to be two orders of magnitude stronger than in any known lanthanide system. This is of the same order of magnitude as the crystal-field splitting of *J*-multiplets on the lanthanide sites, implying that the picture of exchange interaction involving individual crystal-field doublets, Eq. (1), is no longer valid for these compounds. Moreover, the terbium complex from this series exhibits a magnetic hysteresis at 14 K and a 100 s blocking time at 13.9 K (one of the highest blocking temperatures among existing SMMs^24^), suggesting a possible implication of the giant exchange interaction in this SMM behavior.

The purpose of the present work is to reveal the mechanism of giant exchange interaction and the origin of the magnetization blocking of the series of the complexes based on adequate theoretical treatment. We apply an approach combining *ab initio* and density-functional theory (DFT) calculations with microscopic model description to unravel the nature of this exchange interaction. We also elucidate the origin of blocking barriers in these compounds and discuss the effect of strength of exchange interaction on magnetization blocking in strongly anisotropic complexes.

## Results

### Origin of giant exchange interaction

**Figure 1:**
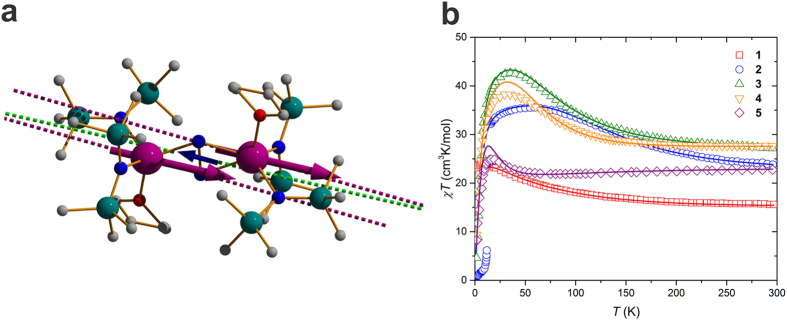
Molecular structure of Tb complex 2 and magnetic susceptibility in the series 1–5. (**a**) Colors’ legend for the balls: violet, Tb; blue, N; red, O; green, Si; grey, C. The hydrogen atoms are omitted for clarity. The violet dashed lines show the orientation of the main anisotropy axes of Tb ions in their ground doublet state, whereas the green dashed line shows the orientation of the main anisotropy axis in the ground exchange Kramers doublet. The violet arrows show the orientation of the local magnetic moments on Tb ions, and the blue arrow on the radical, in the ground exchange Kramers doublet. (**b**) Experimental (symbols) and *ab initio* calculated (lines) temperature-dependent powder magnetic susceptibility (*χ*) for **1**–**5**. The experimental data were upscaled by 3, 3, 1% for **2**, **3** and **5**, respectively, and were downscaled by 2% for **4**. The magnetic susceptibility curves were calculated following the way they have been measured^14^,^24^, as *M*(**H**, *T*)/*H* at *H* = 1 T, averaged over all directions of magnetic field **H** relative to molecular frame. For the computational methodology of the magnetic axes and *χT*, see refs 32 and 33, respectively.

To understand the origin of such strong exchange interaction, we consider the simplest complex of the series, the gadolinium one. In this system the isotropic spins of Gd^3+^ ions (*S*_Gd_ = 7/2) interact with the radical spin of the 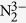 bridge 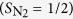 via Heisenberg exchange interaction, 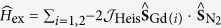, described by a single parameter 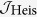 due to the inversion symmetry of the complex (Fig. 1a). Broken-symmetry DFT calculations^25^ give the value 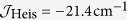 in close agreement with the experimental one, 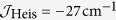
14, and the previous DFT calculations^26^,^27^.

**Figure 2:**
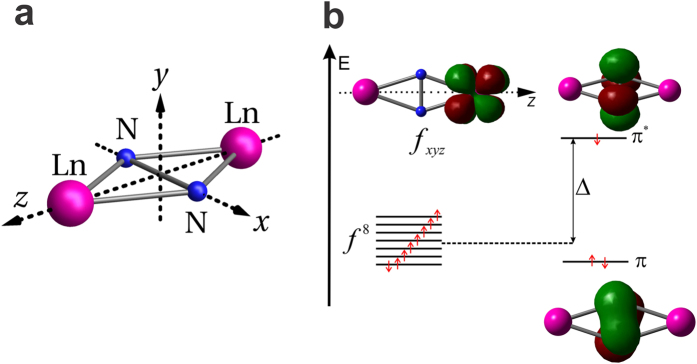
(**a**) Exchange core Ln^3+^-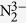-Ln^3+^ in the complex corresponding to *D*_2*h*_ symmetry. (**b**) Magnetic orbitals in **1** obtained from DFT calculations. Only the *f* orbital involved in the kinetic exchange mechanism is shown.

To get insight into the mechanism responsible for the obtained huge value of 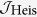, we projected a series of DFT calculations into the effective tight-binding and Hubbard models acting in the space of interacting magnetic orbitals of two Gd ions and the radical (see the Supplemental Material for details). Because of the *D*_2*h*_ symmetry of the exchange core (Fig. 2a), the antibonding π* orbital accommodating the unpaired electron of 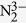 radical overlaps with only one of the 4*f* orbitals on each Ln site, the *xyz* one (Fig. 2b). The corresponding transfer parameter *t* was derived for the Gd complex as *t* = 1407 cm^−1^. The value of *t* is obtained large because the radical’s magnetic orbital π* resides on nearest-neighbor atoms (nitrogens) to both lanthanides. Most important, this orbital is found to lie higher than the 4*f*_*xyz*_ orbitals by as much as Δ = 5.2 × 10^4^ cm^−1^ (Fig. 2b). Because of this huge energy gap, small electron promotion energy is expected for the electron transfer from the π* to the 4*f*_*xyz*_ orbitals: the Coulomb repulsion energy between the transferred electron and the *f* electrons is cancelled at large extent by Δ. On the other hand, because of the same large gap Δ, the promotion energy of electron transfer from 4*f* to π* orbital is at least one order of magnitude larger. Therefore, the contribution of this process to the exchange coupling can be neglected. Indeed, our analysis using the Hubbard model gives the experimental 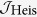 for the Gd complex with (averaged) promotion energy of 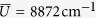, a value many times smaller than typical “Hubbard *U*” in metal complexes^28^. Taking into account only the dominant virtual electron transfer, (4*f*)^7^ (π*)^1^ → (4*f*)^8^ (π*)^0^ → (4*f*)^7^ (π*)^1^, the kinetic contribution to the Gd^3+^-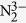
exchange parameter is written in a good approximation as 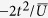
29,30.

Compared to this mechanism, the other contributions such as the direct exchange, the delocalization of unpaired electron of 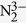 into the empty 5*d* orbitals of Gd^3+^ (Goodenough’s mechanism^31^), the spin polarization and the magnetic dipolar interaction between Gd^3+^ ions are expected to be 1–2 orders of magnitude smaller. The reason is that all these contributions are expected to be of the same order of magnitude as in other lanthanide-radical compounds. Indeed, the direct exchange integral depends only on the shape of the 4*f* an radical’s orbitals, which is not expected to be much different from other complexes. The Goodenough’s contribution arises from higher (third) order of the perturbation theory compared to the usual kinetic exchange, and involves the excitation energy into a higher 5*d* orbital on the Ln site. Both these contributions are usually neglected unless the conventional kinetic exchange appears to be small^29^,^30^. The spin polarization mechanism starts to play a role when the ligand bridging the magnetic centers contains a spectrum of low-lying orbital excitations, which is certainly not the case of 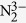. As for magnetic dipolar interaction, it is estimated for Gd^3+^-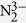 to be ~0.25 cm^−1^.

The same physical situation is realized in the other complexes of the series. As Table 1 shows, the transfer parameters only slightly decrease with the increase of Ln atomic number. On the other hand, the gap Δ between the 4*f* and the π* orbital levels is obtained as huge as in the Gd complex (Table 1), leading again to small promotion energy and, consequently, to the dominant role of the kinetic mechanism in the Ln^3+^-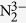 exchange coupling of complexes **2**–**5**. Given the small change of *t* through **1**–**5**, the strong variation of the strength of exchange interaction in this series of complexes, testified by the experimental magnetic susceptibilities (Fig. 1b), is expected to be due to the variation of the promotion energy.

### Anisotropic exchange interaction

Contrary to the Gd complex, the other members of the series are characterized by strong magnetic anisotropy on the Ln sites induced by the crystal-field (CF) splitting of their atomic *J* multiplets. These CF-split multiplets are described by multi-configurational wave-functions, therefore, they should be treated by explicitly correlated *ab initio* approaches^32^,^33^ rather than DFT. The *ab initio* fragment calculations show that the CF split *J* multiplet on Tb^3+^ ions (≈700 cm^−1^) is of the same order of magnitude as the estimated isotropic exchange splitting in **1** (≈400 cm^−1^). Therefore, in sharp contrast with the common situation in lanthanides, the exchange coupling in the anisotropic **2**–**5** does not reduce to the interaction between individual (lowest) CF doublets on Ln sites with the *S* = 1/2 spin of the radical, Eq. (1), but will intermix the entire CF spectrum arising from the ground atomic *J* multiplet at lanthanide ions. Then, such exchange interaction should be formulated in terms of the total angular momenta 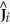 (*i* = 1, 2) on the lanthanide sites.

Extending the Anderson’s superexchange theory^29^,^30^ to strong spin-orbit coupled systems, the tensorial form of the kinetic (covalent) interaction has been recently derived from the microscopic electronic Hamiltonian^34^. The kinetic interaction between the lanthanide and radical centers contains besides the exchange part 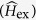 also the Ln^3+^-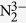 covalent contribution (arising from Ln^3+^-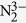 electron delocalization) to the CF splitting at the Ln^3+^ sites 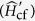:


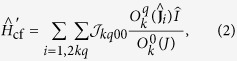



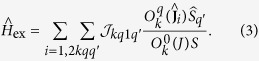


Here, 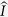 and 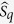 are the unit and the spin operators, respectively, of the radical’s spin *S* = 1/2, 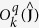 are the Stevens operators^35^ of rank *k* and component *q*, and 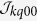 and 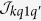 are the exchange parameters^34^. The Stevens operator 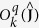 is a polynomial of 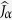 (*α* = *x*, *y*, *z*) of *k*th degree, in which |*q*| (=±*q*) corresponds to the order of 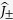 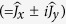. The maximal rank of *k* is 7 for the considered Ln^3+^ ions, whereas the maximal |*q*| is 5 in the present case because only the 4*f*_*xyz*_ magnetic orbitals at the lanthanide sites contribute to the kinetic exchange. The summation over *k* in Eqs (2) and (3) is confined to even and odd ranks, respectively, which is required by the invariance of these Hamiltonians with respect to time-inversion. As it is seen from the form of these Hamiltonians, 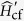 only contributes to the CF splitting of *J* multiplets on individual metal sites, whereas 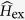 describes the interaction between powers of total angular momenta at the metal sites with components of spin *S* = 1/2 of the 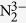 radical. For comparison, the weak anisotropic exchange interaction between two spins (pseudospins) is described by the exchange Hamiltonian 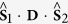, where **D** is the 3 × 3 exchange matrix, containing one isotropic, five symmetric anisotropic and three antisymmetric (Dzyaloshinsky-Moriya) exchange parameters^36^. This Hamiltonian corresponds to the first rank contribution (*k* = 1) in Eq. (3), where 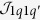 are just the nine components of the above exchange matrix **D**. The expression for the exchange parameter 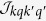
34 includes all virtual electron transfer processes, (4*f*)^*n*^ (π*)^1^ → (4*f*)^*n*+1^ (π*)^0^ → (4*f*)^*n*^ (π*)^1^, where *n* is the number of 4*f* electrons in Ln^3+^. The multiplet electronic structure of Ln^2+^ is fully included in the electron promotion energy *U*_0_ + Δ*E*_*α*_ and the wave functions of the intermediate states, where by *U*_0_ we further denote the smallest promotion energy, *α* numbers the intermediate *J*-multiplets, and Δ*E*_*α*_ is the excitation energy of the multiplet *α* with respect to the ground one in Ln^2+^.

The highly complex tensorial form of the exchange Hamiltonian is inevitable for orbitally degenerate systems with strong spin-orbit coupling, as was pointed out long time ago^37^,^38^. Although all exchange parameters 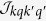 are in principle required for adequate description of the exchange interaction, it is hardly possible to extract a sufficient large number of them from experiment in a unique way. However, once 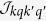 are expressed via microscopic electronic parameters^34^, the latter can be determined from up-to-date quantum chemistry calculations. Thus the transfer parameter *t* is obtained here from DFT calculations, expected to be accurate enough^39^,^40^, whereas the excitation energies Δ*E*_*α*_ and the CF states are obtained by fragment state-of-the-art *ab initio* calculations including spin-orbit coupling^32^,^33^. The only parameter that might be inaccurate when extracted from DFT or *ab initio* calculations is *U*_0_. Indeed, the former gives at most an averaged value over multiplets 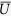 and the latter systematically overestimates it due to insufficient account of dynamical correlation.

In this way we construct the full microscopic Hamiltonian, 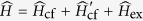, containing only one unknown parameter *U*_0_, where 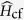 is the *ab initio* CF Hamiltonian for mononuclear Ln fragments (see Supplemental Materials for details). Diagonalizing this Hamiltonian, the magnetic susceptibility *χ* for the entire series of compounds has been simulated as described elsewhere^33^. Figure 1b shows that the experiment is well reproduced for the values of minimal promotion energy *U*_0_ listed in Table 1. The calculated exchange parameters for the series of the complexes are shown in Table 2. We can see from the table that the exchange interaction involves non-negligible contributions up to the rank *k* = 7.

**Figure 3:**
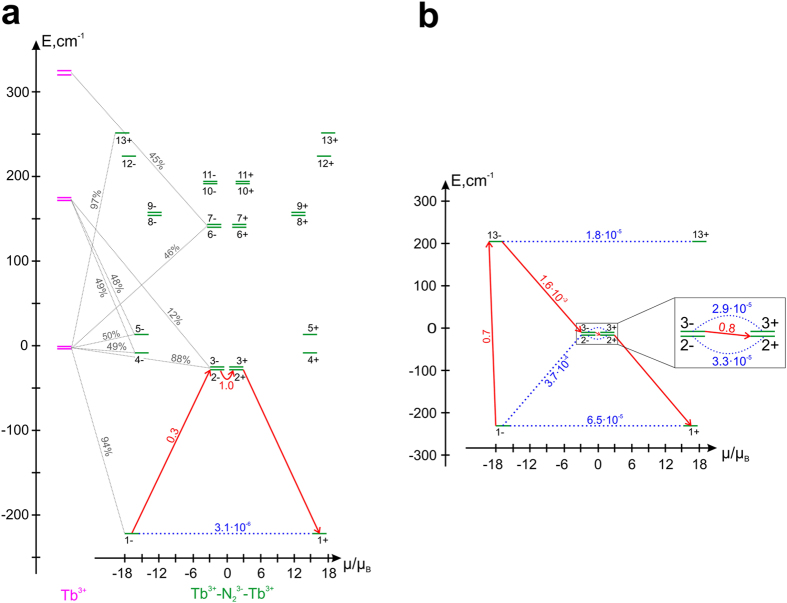
The low-lying exchange spectrum and the magnetization blocking barrier in 2. (**a**) The violet bold lines show the CF levels on Tb ions, the green bold lines show the low-lying exchange levels. Each exchange level is placed according to the projection of its magnetic moment on the main magnetic axis of the ground exchange doublet (green dashed line in Fig. 1a). The exchange levels with the same number are two components of the corresponding KD. The thin dashed lines show the admixed CF states on Tb sites to the exchange states in percent (only admixtures >10% are shown). The number accompanying the blue line is the average magnetic moment matrix element (in *μ*_B_) between the components of the lowest exchange KD; the rate of QTM in the ground exchange state is proportional to its square. The red arrows denote the relaxation path outlining the barrier of reversal of magnetization, with the same meaning of the corresponding numbers (see the text for more details). (**b**) The magnetization blocking barrier for **2** calculated in the absence of the admixture of excited CF states on Tb sites to the ground one via the exchange interaction.

The low-lying exchange spectrum for the Tb complex is shown in Fig. 3a. The ground (1±) and the first two excited (2±, 3±) exchange Kramers doublets (KDs) mainly originate from the ground CF doublets on the Tb ions (94%, 87%, and 88%, respectively). However, the third and fourth excited exchange KDs (4±, 5±) represent almost equal mixtures of the ground and the first excited CF doublets on the Tb^3+^ sites. This is remarkable because the mixed CF states are separated by 166 cm^−1^ (Fig. 3a). Similar scenario is realized in **3** and **4**, whereas in **5** the exchange interaction and the resulting mixing of CF states is relatively weak. The magnetic structure of the ground exchange KD is shown in Fig. 1a. The magnetic moments on Tb^3+^ sites are parallel due to inversion symmetry and almost coincide with the directions of the main magnetic axes in the ground local KDs (Fig. 1a). The magnetic moment of the radical, corresponding to isotropic *S* = 1/2, is rotated with respect to the magnetic moments on Tb sites by small angle *θ* (Table 1) due to the non-Heisenberg contributions to the exchange interaction^23^.

One may notice that the dominant first rank term of the exchange interaction is of isotropic Heisenberg type despite the strong spin-orbit coupling in Ln^3+^ ions (Table 2). This looks surprising because even weak spin-orbit coupling makes the first-rank exchange interaction anisotropic^36^. The analysis of the expression for the first-rank exchange parameters 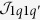
34 shows that they are in general of non-Heisenberg type, whereas the present case is the only possible exception (see Supplemental Material). Indeed, the isotropy of the first-rank exchange contribution requires involvement of only *f* orbitals with the projections *m* = ±2. This can only arise for high symmetry of the exchange bridge (Fig. 2a) and for situations with one single electron transfer path, as in the present case. If any other orbital (or more of them) contribute to the electron transfer, the first-rank exchange interaction becomes strongly anisotropic.

### Magnetization blocking barriers

Table 1 shows that the transverse *g*-factors (*g*_*x*_ and *g*_*y*_) in the ground exchange KD, the squares of which characterize the rate of quantum tunneling of magnetization (QTM)^21^, are the largest for **4** and the smallest for **2** and **3** complexes. This explains why large magnetization hysteresis is seen at low temperatures in the latter two compounds, while not seen at all in the former and only weakly observed in the complex **5** 14,24. The path characterizing the activated magnetic relaxation in high-temperature domain is shown for the Tb complex in Fig. 3a by red arrows. The height of the activation barrier *E*_barrier_ corresponds to the first excited exchange KD, because its two components (2± in Fig. 3a) are connected by a large magnetic moment matrix element which causes a large temperature-assisted QTM. Blocking barriers of similar structure (Fig. 3a) arise in **3** and **4**, their calculated activation energies comparing well with the experimental ones (Table 1).

The unusually large matrix elements between the ground and the first excited exchange KDs are entirely due to the exchange mixing of the ground and the first excited CF doublets on the Ln sites. Indeed, if one quenches the exchange admixture of excited CF doublets to the ground ones, this matrix element becomes three orders of magnitude smaller (Fig. 3b). Then the activated relaxation will proceed via a higher exchange doublet, thereby doubling the height of the blocking barriers (Fig. 3b). Thus in the case of very strong exchange interaction, which is able to intermix the CF states on Ln sites, the axiality of the ground and excited exchange doublets is diminished dramatically and the blocking barriers do not exceed the energy of the first excited exchange KD. In other words, the strength of exchange interaction after reaching a certain value starts playing a destructive role for the magnetization blocking. Therefore, to exploit the effect of strong exchange interaction for achieving high magnetization blocking, an even stronger axial CF field on the Ln sites, precluding the exchange admixture of excited CF states, seems to be indispensable.

## Discussion

The mixed lanthanide complexes **1**–**5** investigated in this work are unique because they show an exchange interaction up to two orders of magnitude stronger than in conventional lanthanide complexes. Due to such strong exchange interaction, a qualitatively new situation arises when the exchange coupling starts to intermix the CF multiplets on the Ln sites. In all previous lanthanide complexes only the ground CF doublets on Ln sites were involved, which led to conventional Ising-type exchange interactions. In the present case, due to the involvement of all CF doublets belonging to the atomic *J*-multiplet, the exchange interaction becomes highly complex, requiring a tensorial description and involving many parameters.

By combining DFT and *ab initio* calculations with the microscopic modeling of the exchange interaction, we were able to unravel the mechanism of giant exchange interaction in these complexes. This exchange interaction is found to be kinetic and highly complex, involving non-negligible contributions up to seventh power of total angular momentum of each Ln site. Based on the calculated exchange states, the mechanism of the magnetization blocking is revealed. Contrary to general expectations the latter is not always favored by strong exchange interaction. The accuracy of our approach is proved by the close reproduction of experimental magnetic susceptibility and magnetization blocking barrier for all investigated compounds.

The theoretical analysis proposed in this work opens the way for the investigation of highly complex exchange interaction in materials with strongly anisotropic magnetic sites. Given the large number of involved exchange parameters and the obvious difficulties of their experimental determination, such an approach can become a powerful tool for the study of magnetic materials of primary interest.

## Methods

### DFT calculations

All DFT calculations were carried out with ORCA 3.0.0. program^41^ using the B3LYP functional and SVP basis set. Scalar relativistic effects were taken into account within Douglas-Kroll-Hess Hamiltonian. The isotropic exchange parameter for the complex **1**, 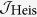, was derived by applying the broken-symmetry approach^25^. The obtained 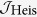 was divided by 2 to account for its overestimation due to the self-interaction error^42^,^43^. The 4*f* and the π* orbital levels and the transfer parameters *t* for all complexes **1**–**5** were derived by projecting the Kohn-Sham orbitals onto a tight-binding model. The averaged promotion energy 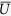 for the complex **1** was derived by reproducing the energy difference between the high-spin and the broken-symmetry DFT states with a Hubbard model.

### *Ab initio* calculations

Energies and wave functions of CF multiplets on Ln^3+^ sites in **1**–**5** have been obtained from fragment *ab initio* calculations including the spin-orbit coupling, using the quantum chemistry package Molcas 7.8^44^. The calculations have been done for the experimental geometry of the complexes, in which one of the two Ln^3+^ ions was replaced by an isovalent closed-shell La^3+^ ions. The total number of electrons was reduced by unity in order to have a closed-shell electronic configuration 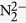 on the dinitrogen bridge. To simulate the electrostatic crystal field from the removed radical’s electron, two point charges of −0.5*e* were added on the nitrogen atoms. For this structural model of a Ln fragment, the complete active space self-consistent field (CASSCF) approach was used including all seven 4*f* orbitals of the Ln atom in the active space. The spin-orbit interaction was treated with the module SO-RASSI and the local magnetic properties were calculated with the SINGLE_ANISO module of Molcas^45^. Exchange energy spectrum and magnetic properties of the investigated polynuclear compounds were calculated using the POLY_ANISO program^33^,^45^, modified to treat the general form of exchange interaction, Eqs. (2), (3), within the kinetic exchange mechanism.

For further details, see Supplemental Material.

## Additional Information

**How to cite this article**: Vieru, V. *et al.* Giant exchange interaction in mixed lanthanides. *Sci. Rep.* **6**, 24046; doi: 10.1038/srep24046 (2016).

## Supplementary Material

Supplementary Information

## Figures and Tables

**Table 1:** Transfer parameters *t*, energy gaps Δ between the 4*f* and the π* orbital levels, minimal electron promotion energies *U*_0_ (all in cm^−1^), *g*-factors and angles between the magnetic moments on Ln^3+^ and 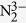 (*θ*) in the ground exchange KD, and blocking barriers *E*_barrier_ (cm^−1^) for complexes 1–5.

	1 (Gd)	2 (Tb)	3 (Dy)	4 (Ho)	5 (Er)
*t*	1407	1333	1322	1311	1270
Δ	5.20 × 10^4^	5.74 × 10^4^	5.80 × 10^4^	5.73 × 10^4^	5.78 × 10^4^
*U*_0_	8500	4600	6500	7400	12200
*g*_*x*_	2.2 × 10^−2^	7.6 × 10^−6^	2.2 × 10^−6^	4.7 × 10^−3^	1.3 × 10^−3^
*g*_*y*_	3.7 × 10^−2^	1.1 × 10^−5^	7.0 × 10^−6^	1.2 × 10^−2^	1.6 × 10^−3^
*g*_*z*_	25.6	33.6	37.5	36.2	32.1
*θ*	0.0°	2.5°	2.3°	2.6°	6.2°
*E*_barrier_ (exp.)	–	227	123	73	36
*E*_barrier_ (calc.)	–	208	121	105	28

For *E*_barrier_, both the experimental (exp.)^14^,^24^ and present (calc.) data are shown.

**Table 2:** Calculated exchange parameters 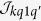 (cm^−1^) for the complexes 1–5.

*k*	*q*	*q*′	J_*kq1q*′_				
			1 (Gd)	2 (Tb)	3 (Dy)	4 (Ho)	5 (Er)
1	0	0	94.9	95.8	70.8	55.4	24.2
1	±1	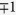	−94.9	−95.8	−70.8	−55.4	−24.2
3	0	0	0.0	13.4	−10.6	−4.4	5.0
3	±1	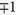	0.0	8.2	−6.5	−2.7	3.0
3	±3	±1	0.0	10.6	−8.4	−3.5	3.9
5	0	0	0.0	17.0	−16.0	−1.6	4.2
5	±1	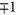	0.0	−12.8	8.4	6.8	−6.1
5	±3	±1	0.0	−2.5	7.5	−7.6	3.4
5	±4	0	0.0	5.7	−0.8	−7.5	5.0
5	±5	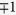	0.0	−13.5	11.5	3.2	−4.4
7	0	0	0.0	0.3	−3.3	4.6	−2.3
7	±1	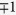	0.0	−0.2	2.5	−3.5	1.7
7	±3	±1	0.0	0.2	−2.2	3.0	−1.5
7	±4	0	0.0	−0.4	5.1	−7.1	3.5
7	±5	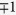	0.0	0.6	−7.2	10.0	−5.0
