# Supplementary material for: Giant exchange interaction in mixed lanthanides
Source: Sci Rep. 2016 Apr 18;6:24046. doi: 10.1038/srep24046 (PMC4834576; doi:10.1038/srep24046)
Supplement: Supplementary Information [file srep24046-s1.pdf]

Supplemental Material
for
"Giant exchange interaction in mixed lanthanides"

Veacheslav Vieru, Naoya Iwahara, Liviu Ungur, and Liviu F. Chibotaru
Theory of Nanomaterials Group, Katholieke Universiteit Leuven,
Celestijnenlaan 200F, B-3001 Leuven, Belgium

(Dated: February 21, 2016)

Abstract

This material contains:

1) DFT based derivations of the $4f$ and the π^* orbital levels and of the transfer parameters t for all complexes **1-5**;

2) Fragments *ab initio* calculations of the energies and wave functions of CF multiplets on Ln^{3+} sites in **1-5**, and calculations of atomic multiplets of the corresponding Ln^{2+} ions;

3) The calculation of the exchange spectra are described;

4) The analysis of the first rank exchange parameters.

I. DFT CALCULATIONS

A. Extraction of the transfer parameter t for 1-5

In order to derive the transfer parameters between the $4f$ orbital and the π^* orbital of the bridging N_2, the Kohn-Sham levels are projected into tight-binding Hamiltonian:

$$\hat{H} = \sum_{\sigma} \left[\sum_{i=1}^{2} \epsilon_f \hat{n}_{i\tilde{\gamma}\sigma} + \epsilon_{\pi^*} \hat{n}_{\pi^*\sigma} + t \left(\hat{c}^\dagger_{1\tilde{\gamma}\sigma} \hat{c}_{\pi^*\sigma} + \hat{c}^\dagger_{\pi^*\sigma} \hat{c}_{1\tilde{\gamma}\sigma} - \hat{c}^\dagger_{2\tilde{\gamma}\sigma} \hat{c}_{\pi^*\sigma} - \hat{c}^\dagger_{\pi^*\sigma} \hat{c}_{2\tilde{\gamma}\sigma} \right) \right], \tag{S1}$$

where $i(= 1, 2)$ is the index for the Ln^{3+} site in the complex, N_2^{3-} site is described by the type of the magnetic orbital π^*, $\tilde{\gamma}$ is the orbital component xyz, $\sigma =\uparrow, \downarrow$ is the projection of spin operator, ϵ_f and $\epsilon_{\pi^*}(= \epsilon_f + \Delta)$ are one electron orbital levels of the $4f$ orbital and the π^* orbital, respectively, t is the transfer parameter between the $4f$ and the π^* orbitals, $\hat{c}^\dagger$ ($\hat{c}$) is an electron creation (annihilation) operator, and $\hat{n}$ is a number operator. The subscripts of the creation, annihilation, and number operators indicate the site, the orbital index for only lanthanide site, and spin projection. Because of the D_{2h} symmetry of the magnetic core part, only one $4f$ orbital ($4f_{xyz}$) overlaps with the π^* orbital (Fig. 2b in the main text). Therefore, we only include the $4f_{xyz}$ orbital for each lanthanide site in the model Hamiltonian.

Diagonalizing the tight-binding Hamiltonian (S1), the one-electron levels are obtained as

$$\epsilon_{f,a} = \epsilon_f, \tag{S2}$$

$$\epsilon_{f,s} = \epsilon_f + \frac{1}{2}\left(\Delta - \sqrt{\Delta^2 + 8t^2}\right), \tag{S3}$$

$$\epsilon_{\pi^*} = \epsilon_f + \frac{1}{2}\left(\Delta + \sqrt{\Delta^2 + 8t^2}\right), \tag{S4}$$

where the subscript "a" and "s" indicate antisymmetric and symmetric orbitals, respectively. Comparing these orbital levels with the DFT calculations, we obtain parameters ϵ_f, t, and Δ.

The highest occupied Kohn-Sham orbital for the down spin in the low-symmetry DFT solutions correspond to the π^* orbital. On the other hand, $4f$ atomic orbitals contribute to many Kohn-Sham orbitals. Thus, the $4f$ orbitals are localized as follows. Because of the inversion symmetry of the complexes, the $4f$ orbital part of each Kohn-Sham orbital ψ_i is decomposed into the antisymmetric and symmetric parts:

$$|\psi_i\rangle = \frac{1}{\sqrt{2}}\left(|1\rangle + |2\rangle\right) C_{a,i} + \frac{1}{\sqrt{2}}\left(|1\rangle - |2\rangle\right) C_{s,i}, \tag{S5}$$

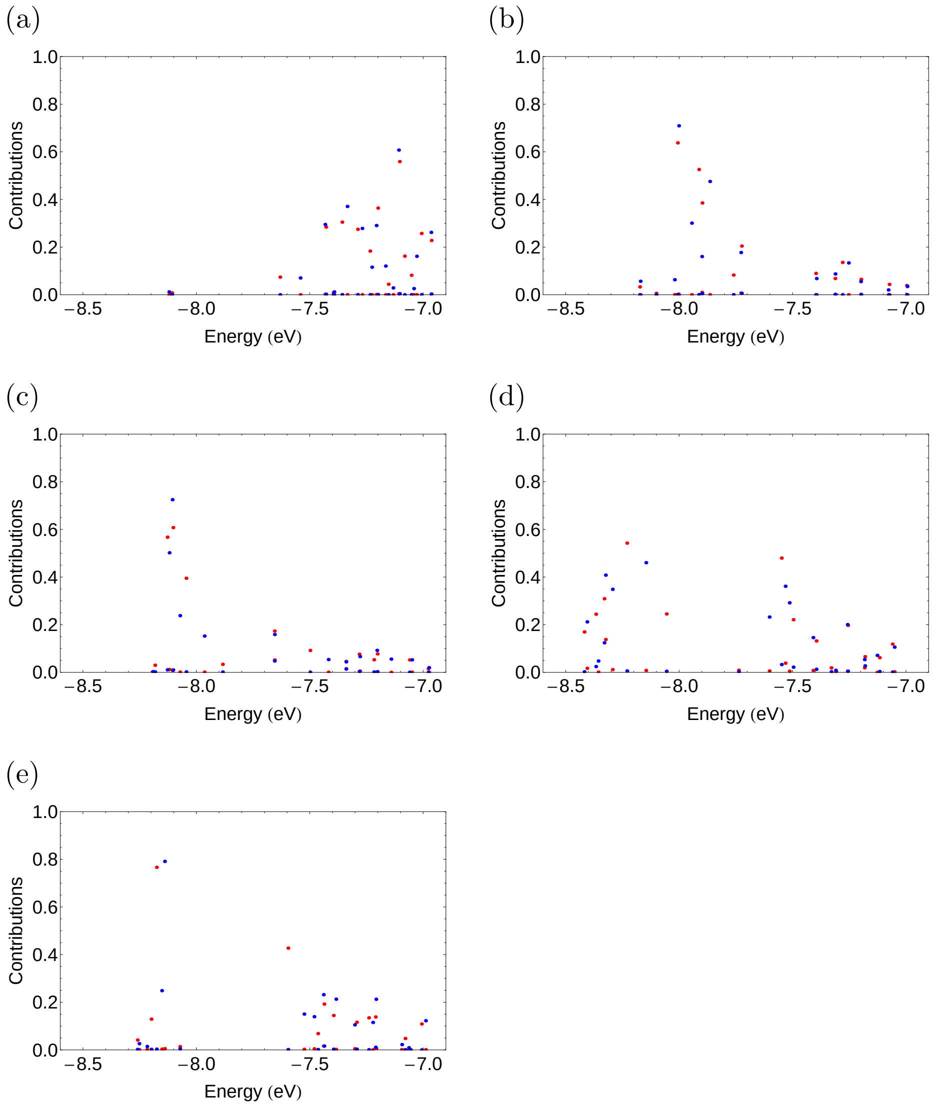

FIG. S1. Contributions of the antisymmetric $|C_{a,i}|$ (red) and the symmetric $|C_{s,i}|$ (blue) combinations of the $4f_{xyz}$ orbitals to each Kohn-Sham orbitals for the (a) Gd, (b) Tb, (c) Dy, (d) Ho, (e) Er complexes.

where, $|1\rangle$ and $|2\rangle$ indicate the $4f_{xyz}$ orbitals on the first and the second lanthanide sites, respectively. The absolute values of $C_{a,i}$ and $C_{s,i}$ for the occupied Kohn-Sham orbitals for the up spin part are shown in Fig. S1. As the antisymmetric and the symmetric levels, we averaged the Kohn-Sham levels:

$$\epsilon_{f,a} = \frac{\sum_i^{\text{occ.}} C_{a,i}^2 \epsilon_i}{\sum_i^{\text{occ.}} C_{a,i}^2}, \qquad \epsilon_{f,s} = \frac{\sum_i^{\text{occ.}} C_{s,i}^2 \epsilon_i}{\sum_i^{\text{occ.}} C_{s,i}^2}. \tag{S6}$$

In Eq. (S6), the sum is taken over occupied Kohn-Sham orbitals. With the use of the levels,

the parameters t and Δ are derived (Table I in the main text). The transfer parameter is gradually decreasing as the increase of the atomic number because the ionic radius of the lanthanide shrinks.

B. Calculation of $\pi^* \to 4f$ electron promotion energy for 1

The high- and low-spin states of the complex **1** were analyzed based on the Hubbard Hamiltonian:

$$\begin{aligned}\hat{H} &= \sum_{i=1,2} \sum_{\gamma\sigma} \epsilon_f \hat{n}_{i\gamma\sigma} + \sum_{\sigma} \epsilon_{\pi^*} \hat{n}_{\pi^*\sigma} \\ &+ \sum_{\sigma} t \left(\hat{c}^\dagger_{1\tilde{\gamma}\sigma} \hat{c}_{\pi^*\sigma} + \hat{c}^\dagger_{\pi^*\sigma} \hat{c}_{1\tilde{\gamma}\sigma} - \hat{c}^\dagger_{2\tilde{\gamma}\sigma} \hat{c}_{\pi^*\sigma} - \hat{c}^\dagger_{\pi^*\sigma} \hat{c}_{2\tilde{\gamma}\sigma} \right) \\ &+ \sum_{i=1,2} \sum_{\langle \gamma\sigma, \gamma'\sigma' \rangle} u_f \hat{n}_{i\gamma\sigma} \hat{n}_{i\gamma'\sigma'} + u_{\pi^*} \hat{n}_{\pi^*\uparrow} \hat{n}_{\pi^*\downarrow} + \sum_{i=1,2} \sum_{\gamma\sigma} \sum_{\sigma'} v \hat{n}_{\gamma\sigma} \hat{n}_{\pi^*\sigma'}, \end{aligned} \quad \text{(S7)}$$

where γ is the component of the $4f$ orbital, u_f and u_{π^*} are the intrasite Coulomb repulsions on Gd and N_2 sites, respectively, and v is the intersite Coulomb repulsion between the Gd and N_2 sites.

The high-spin state with the maximal projection is described by one electron configuration:

$$|1 \uparrow, \pi^* \uparrow, 2 \uparrow\rangle, \quad \text{(S8)}$$

where 1 and 2 are the lanthanide sites and $\uparrow$ and $\downarrow$ are spin projections. The $4f$ electrons which are not in the $4f_{xyz}$ orbital are not explicitly written here. The total energy E_{HS} is

$$E_{\mathrm{HS}} = E_0 + (2n+1)\epsilon_f + \Delta + 2nv + n(n-1)u_f, \quad \text{(S9)}$$

where E_0 is the total electronic energy except for the electrons in the $4f$ orbitals and π^* orbitals, and n is the number of the $4f$ electrons in Gd^{3+} ion. For the low-spin state ($\uparrow, \downarrow, \uparrow$ type), the basis set is

$$\{|1 \uparrow, 1 \downarrow, 2 \uparrow\rangle, |1 \uparrow, \pi^* \downarrow, 2 \uparrow\rangle, |1 \uparrow, 2 \downarrow, 2 \uparrow\rangle\}. \quad \text{(S10)}$$

Here, the configurations with the electron transfer from the $4f$ to the π^* are not included because these configurations do not contribute much to the low-energy states due to the large energy gap Δ between the $4f$ and the π^* levels. The lowest energy is

$$E_{\mathrm{LS}} = E_0 + (2n+1)\epsilon_f + n(n-1)u_f + \frac{1}{2}\left(\Delta + 2nv + nu_f - \sqrt{(\Delta + 2nv - nu_f)^2 + 8t^2} \right). \quad \text{(S11)}$$

The energy difference between the low- and high-spin states are

$$\Delta E = E_{\mathrm{LS}} - E_{\mathrm{HS}} \tag{S12}$$
$$= \frac{1}{2}\left[nu_f - (\Delta + 2nv) - \sqrt{(\Delta + 2nv - nu_f)^2 + 8t^2}\right] \tag{S13}$$
$$= \frac{1}{2}\left(\bar{U} - \sqrt{\bar{U}^2 + 8t^2}\right), \tag{S14}$$

where

$$\bar{U} = nu_f - \Delta - 2nv \tag{S15}$$

is the (averaged) electron promotion energy. Eq. (S15) shows that (i) the energy gap Δ significantly reduces the promotion energy and (ii) the promotion energy increases with the number of the $4f$ electrons n. Using the transfer parameter t derived from the Kohn-Sham orbital, energy gaps between the high-spin state and low-spin state, and Eq. (S14), the averaged promotion energy $\bar{U}$ is derived.

II. *AB INITIO* CALCULATIONS

A. Fragment calculations for Ln^{3+} centers in 1-5

To obtain the local electronic properties of the magnetic ions, *ab initio* quantum chemistry calculations (CASSCF/SO-RASSI) were performed using Molcas [1]. In the calculations, one of the metal ions in the complex was replaced by diamagnetic lanthanum ion (La^{3+}) and the ligands for the La ion were reduced (Fig. S2). Two point charges (-0.5 e) were put on each N atom creating the N_2 bridge, where e (> 0) is the elementary charge. The latter is to include the electrostatic potential from the unpaired electron of N_2^{3-} bridge. The covalent effect is included later ($\hat{H}'_{\mathrm{cf}}$ in the main text). In the CASSCF calculations, all $4f$ orbitals of the magnetic site are included in the active orbitals. The spin-orbit coupling is included in the SO-RASSI calculation. In the SO-RASSI calculations the following CASSCF states were mixed by spin-orbit coupling: for Gd, 1 octet, 48 sextet, 120 quartet and 113 doublet states, for Tb, 7 septet, 140 quintet, 113 triplet and 123 singlet states, for Dy, 21 sextet, 128 quartet and 130 doublet states, for Ho, 35 quintet, 210 triplet and 196 singlet states, and for Er, 35 quartet and 112 doublets states. As the basis set for the calculations, ANO-RCC was used. The contraction of the basis set is shown in Table S1. The Cholesky

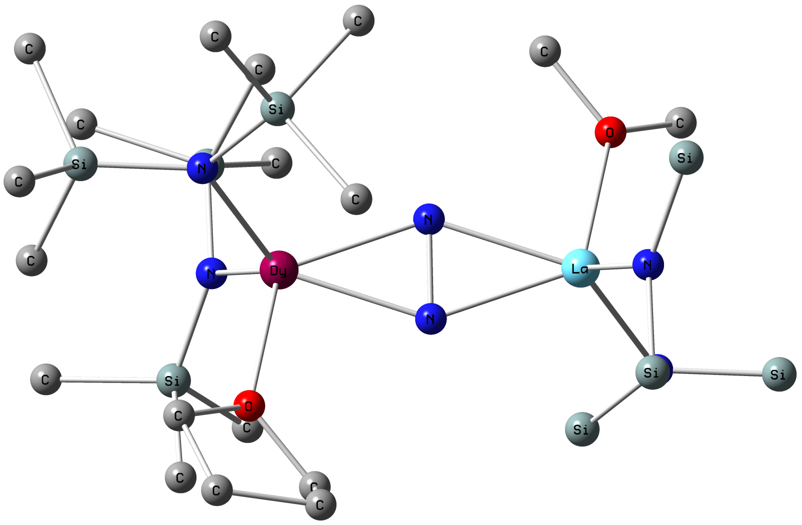

FIG. S2. The $LnLaN_2^{3-}$ fragment used in *ab initio* calculations. Hydrogen atoms are omitted for clarity. The right lanthanide ion was replaced by La in the *ab initio* calculations.

TABLE S1. Contractions of the employed ANO-RCC basis sets for the *ab initio* calculations.

Ln	7s6p4d2f1g	Si	4s3p
La	7s6p4d2f	O	3s2p
N (N_2 bridge)	3s2p1d	C	3s2p
N (the others)	3s2p	H	2s

decomposition threshold was set to 5×10^{-8} Hartree. The obtained SO-RASSI wave functions were transformed into pseudo spin states (or pseudo $\tilde{J}$ states) [2–5] to analyze the magnetic data using SINGLE_ANISO module [6].

The obtained crystal-field (CF) levels are shown in Table S2. In all cases, the lowest spin-orbit states are doubly degenerate (Kramers doublet (KD) for Ln = Gd, Dy, Er) or quasidegenerate (Ising doublet for Ln = Tb, Ho). The ground CF states $|\psi\rangle$ are decomposed into the sum of the ground pseudo $\tilde{J}$ multiplets $|JM\rangle$ [4, 5]:

$$|\psi\rangle = \sum_{M=-J}^{J} C_M |JM\rangle. \tag{S16}$$

The coefficients C_M are shown in Table S3. The contributions of the multiplets with the largest projection ($|M| = J$) to the ground CF states are 94.2 %, 96.4 %, 97.1 %, 91.7 %, 78.6 %, for Gd, Tb, Dy, Ho, and Er, respectively. For each ground doublets, the g-tensors

TABLE S2. The lowest spin-orbit levels of Ln centers obtained by *ab initio* fragment calculations (cm^{-1}). The covalency effect is not included.

Gd	Tb	Dy	Ho	Er
0.000	0.000	0.000	0.000	0.000
0.000	0.099	0.000	0.982	0.000
0.329	141.153	179.143	87.999	74.691
0.329	142.222	179.143	88.534	74.691
0.631	288.590	320.747	130.818	118.034
0.631	295.694	320.747	147.454	118.034
1.108	401.446	406.717	167.052	166.279
1.108	435.925	406.717	202.496	166.279
	490.539	470.573	224.559	212.344
	531.201	470.573	241.625	212.344
	547.372	531.942	246.712	262.700
	730.715	531.942	284.704	262.700
	731.087	623.187	296.344	295.345
		623.187	323.988	295.345
		749.919	327.012	396.290
		749.919	385.730	396.290
			386.659	

are calculated (Table S4). The Er complex is not magnetically anisotropic as much as the other complexes (Tb, Dy, Ho). This is because the multiplets $|JM\rangle$ with small M ($|M| < J$) are mixed more than the other systems.

B. Calculation of atomic J-multiplets of Ln^{2+} ions

The excitation energies of the intermediate virtual electron transferred states were replaced by the excitation energies for isolated Ln^{2+} ion (Ln = Gd, Tb, Dy, Ho, Er). To obtain the energies, the CASSCF and the SO-RASSI calculations were performed with ANO-RCC QZP basis set [1]. As in the case of the fragment calculations, all $4f$ orbitals are

TABLE S3. $|JM\rangle$ structure of ground CF doublet on Ln^{3+} center in **1-5**

Gd		Tb		Dy		Ho		Er	
M	$\lvert C_M \rvert$	M	$\lvert C_M \rvert$	M	$\lvert C_M \rvert$	M	$\lvert C_M \rvert$	M	$\lvert C_M \rvert$
−7/2	0.971	−6	0.694	−15/2	0.986	−8	0.677	−15/2	0.887
−5/2	0.001	−5	0.005	−13/2	0.019	−7	0.005	−13/2	0.112
−3/2	0.225	−4	0.123	−11/2	0.164	−6	0.162	−11/2	0.321
−1/2	0.004	−3	0.014	−9/2	0.027	−5	0.044	−9/2	0.168
1/2	0.077	−2	0.024	−7/2	0.023	−4	0.084	−7/2	0.214
3/2	0.002	−1	0.008	−5/2	0.007	−3	0.056	−5/2	0.103
5/2	0.038	0	0.009	−3/2	0.010	−2	0.039	−3/2	0.105
7/2	0.000	1	0.008	−1/2	0.004	−1	0.033	−1/2	0.024
		2	0.024	1/2	0.002	0	0.027	1/2	0.031
		3	0.014	3/2	0.001	1	0.033	3/2	0.021
		4	0.123	5/2	0.001	2	0.039	5/2	0.011
		5	0.005	7/2	0.000	3	0.056	7/2	0.012
		6	0.694	9/2	0.000	4	0.084	9/2	0.016
				11/2	0.000	5	0.044	11/2	0.002
				13/2	0.000	6	0.162	13/2	0.005
				15/2	0.000	7	0.005	15/2	0.000
						8	0.677		

treated as the active orbitals of the CASSCF calculations. In the SO-RASSI calculations, the following LS terms are included: ^7F for Gd^{2+}, ^6P, ^6F, ^6H for Tb^{2+}, ^5D, ^5F, ^5G, ^5I for Dy^{2+}, ^4F, ^4G, ^4I for Ho^{2+}, and ^3F, ^3H for Er^{2+}. The excitation energies ΔE are shown in Table S5.

TABLE S4. The g tensors for the lowest doublets of Ln centers obtained from the fragment calculations. The transverse g-factors for Tb and Ho are zero because of the Griffith's theorem [7].

	Gd	Tb	Dy	Ho	Er
g_X	0.492	0.000	0.0026	0.000	0.163
g_Y	0.824	0.000	0.0040	0.000	0.227
g_Z	13.439	17.675	19.6459	19.422	16.528

III. ANALYSIS OF FIRST RANK EXCHANGE PARAMETERS

As shown in Table II in the main text, the first rank part ($k = k' = 1$) of the exchange interaction is isotropic Heisenberg type in all complexes, i.e.,

$$\mathcal{J}_{1\pm11\mp1} = -\mathcal{J}_{1010} \neq 0, \tag{S17}$$

and the other $\mathcal{J}_{1q1q'}$ are zero. The reason can be understood analyzing the formula of the exchange interaction. The exchange parameter between J multiplet and isotropic spin 1/2 (Eqs. (2), (3) in the main text) is written as [8]

$$\mathcal{J}_{kqk'q'} = \sum_x \sum_{\alpha_J J} \frac{\{t \times t\}^x_{kqk'q'} \mathcal{G}^1_{\alpha_J J k' x k} \tilde{\mathcal{F}}^2_{k'}}{U_0 + \Delta E^{n+1}_{\alpha_J J}}, \tag{S18}$$

where

$$\{t \times t\}^x_{kqk'q'} = (-1)^{l_1 - k' + q'} \sum_{mm'} \sum_{\xi} t^{12}_{m\pi^*} t^{21}_{\pi^* m'} C^{x\xi}_{l_1 m' kq} C^{x\xi}_{k' -q' l_1 m}, \tag{S19}$$

$t_{m\pi^*}$ is the electron transfer between the $4f$ with component m of orbital angular momentum and the π^* orbital of N_2, $l_1 = 3$ is the magnitude of the atomic orbital angular momentum for f orbital, x ($l_1 - k' \leq x \leq l_1 + k'$) indicates a rank, $\xi = -x, -x+1, ..., x$, $C^{x\xi}_{l_1 m' kq}$ and $C^{x\xi}_{k' -q' l_1 m}$ are Clebsch-Gordan coefficients [9], α_J and J are the LS-term and the total angular momentum of Ln^{2+}, respectively, $\Delta E^{n+1}_{\alpha_J J}$ is the excitation multiplet energies of Ln^{2+}, and $\mathcal{G}^{\mathrm{Ln}}_{\alpha_J J k' x k}$ and $\tilde{\mathcal{F}}^{\mathrm{N}_2}_{k'}$ are functions of their subscripts. For the detailed description of x, $\mathcal{G}^{\mathrm{Ln}}_{\alpha_J J k' x k}$, and $\tilde{\mathcal{F}}^{\mathrm{N}_2}_{k'}$, see Ref. 8.

Since the dependence of the exchange parameter (S18) on q and q' appears only in $\{t \times t\}^x_{kqk'q'}$ (S19), the condition for the isotropy of $\mathcal{J}_{kq1q'}$ is revealed from the equation. First, we consider the cases where only the transfer between $f_{\pm m_0}$ orbitals ($m_0 = 0, 1, 2, 3$) and the

TABLE S5. Excitation energies with respect to the lowest J multiplet of isolated Ln^{2+} ions (meV).

	Gd			Tb			Dy	
LS term	J	ΔE	LS term	J	ΔE	LS term	J	ΔE
^7F	6	0.000	^6H	15/2	0.000	^5I	8	0.000
	5	182.104		13/2	307.374		7	458.212
	4	333.856		11/2	573.765		6	859.148
	3	455.259		9/2	799.172		5	1202.808
	2	546.311		7/2	983.596		4	1489.190
	1	607.012		5/2	1127.038	^5G	6	3412.346
	0	637.362	^6F	11/2	1050.937		5	3756.005
				9/2	1276.345		4	4042.388
				7/2	1460.769	^5F	5	2369.357
				5/2	1604.210		4	2655.740
			^6P	7/2	4357.932	^5D	4	5667.150
				5/2	4501.373			
	Ho			Er				
LS term	J	ΔE	LS term	J	ΔE			
^4I	15/2	0.000	^3H	6	0.000			
	13/2	638.260		5	850.945			
	11/2	1191.419		4	1197.911			
	9/2	1659.477	^3F	4	1560.065			
^4G	11/2	3521.812						
	9/2	3989.870						
^4F	9/2	2461.643						

isotropic spin is nonzero for simplicity. The values of $\{t \times t\}^x_{1q1q'}$ are tabulated in Table S9. We find that the condition (S17) is fulfilled when $m_0 = 2$, while it is not for other m_0. In the case of $m_0 = 1$, the nonzero terms with $q = q' = \pm 1$ are also the source of the anisotropic exchange. When more than one set of f orbitals m_0 contribute to the electron transfer, the exchange interaction becomes always anisotropic. Finally, since Eq. (S19) is independent of

TABLE S6. Kinetic contributions to the CF parameters $\mathcal{J}_{kq00}$ (cm^{-1}) for complexes **1-5**.

k	q	$\mathcal{J}_{kq00}$				
		Gd	Tb	Dy	Ho	Er
0	0	−94.88	−95.77	−70.78	−55.38	−24.20
4	0	5.86×10^{-3}	−30.11	23.76	10.00	−11.15
4	±4	3.50×10^{-3}	−18.00	14.20	5.97	−6.66
6	0	4.12×10^{-7}	-4.95×10^{-1}	6.13	−8.53	4.29
6	±4	-7.70×10^{-7}	9.27×10^{-1}	−11.46	15.96	−8.02

ions, the condition given above applies to the exchange interaction between any f electron ions and spin 1/2.

[1] F. Aquilante, L. De Vico, N. Ferré, G. Ghigo, P.-å. Malmqvist, P. Neogrády, T. B. Pedersen, M. Pitoňák, M. Reiher, B. Roos, *et al.*, "Molcas 7: The next generation," J. Comput. Chem. **31**, 224–247 (2010).

[2] L. F. Chibotaru and L. Ungur, "Ab initio calculation of anisotropic magnetic properties of complexes. I. Unique definition of pseudospin Hamiltonians and their derivation," J. Chem. Phys. **137**, 064112 1–22 (2012).

[3] L. F. Chibotaru, "Ab initio methodology for pseudospin hamiltonians of anisotropic magnetic complexes," in *Adv. Chem. Phys.*, Vol. 153, edited by S. A. Rice and A. R. Dinner (Johns Wiley & Sons, New Jersey, 2013) pp. 397–519.

[4] L. Ungur and L. F. Chibotaru, "Computational Modelling of the Magnetic Properties of Lanthanide Compounds," in *Lanthanides and Actinides in Molecular Magnetism*, edited by R. Layfield and M. Murugesu (Wiley, New Jersey, 2015) Chap. 6.

[5] L. F. Chibotaru, "Theoretical understanding of Anisotropy in Molecular Nanomagnets," in *Molecular Nanomagnets and Related Phenomena*, Struct. Bond., Vol. 164, edited by Song Gao (Springer Berlin Heidelberg, 2015) pp. 185–229.

[6] L. F. Chibotaru and L. Ungur, "The computer programs SINGLE_ANISO and POLY_ANISO," University of Leuven (2006).

TABLE S7. Energies of the CF multiplets (cm^{-1}) of Ln centers in **1-5** originating from the ground atomic J-multiplet of the corresponding Ln^{3+} ions, calculated with included kinetic contribution. Due to the latter, the ground CF multiplets of Ln centers are stabilized by 95, 110, 58, 55, 14 cm^{-1} for Gd, Tb, Dy, Ho, Er, respectively.

Gd	Tb	Dy	Ho	Er
0.000	0.000	0.000	0.000	0.000
0.000	0.055	0.000	1.297	0.000
0.329	168.191	163.450	95.958	68.276
0.329	168.965	163.450	96.064	68.276
0.630	316.136	300.460	129.220	112.297
0.630	318.418	300.460	147.698	112.297
1.108	426.550	396.451	174.642	150.837
1.108	444.805	396.451	211.117	150.837
	501.172	458.147	223.439	197.730
	541.183	458.147	248.194	197.730
	556.192	510.902	250.839	249.831
	740.636	510.902	280.160	249.831
	741.002	604.121	290.136	276.581
		604.121	320.445	276.581
		747.503	322.767	387.217
		747.503	371.677	387.217
			371.948	

[7] J. S. Griffith, "Spin hamiltonian for even-electron systems having even multiplicity," Phys. Rev. **132**, 316–319 (1963).

[8] N. Iwahara and L. F. Chibotaru, "Exchange interaction between J multiplets," Phys. Rev. B **91**, 174438 1–18 (2015).

[9] D. A. Varshalovich, A. N. Moskalev, and V. K. Khersonskii, *Quantum Theory of Angular Momentum* (World Scientific, Singapore, 1988).

TABLE S8. Energy of the low-lying exchange KDs (cm^{-1}) in **1-5**

Gd	Tb	Dy	Ho	Er
0.000	0.000	0.000	0.000	0.000
0.381	207.619	120.686	105.154	27.999
0.643	207.670	120.686	106.791	28.000
0.865	210.623	158.882	108.718	53.467
1.187	227.323	164.275	110.590	64.209
1.605	362.446	252.462	146.181	68.710
2.112	366.170	273.941	153.019	86.241
27.527	369.751	273.943	160.514	86.254
27.761	369.876	293.589	161.003	99.987

TABLE S9. $\{t \times t\}^x_{1q1q'}$ for $m_0 = 0, 1, 2, 3$.

x	q_1	q_2	m_0			
			0	1	2	3
2	0	0	$\frac{3}{7}\lvert t^{12}_{0\pi^*}\rvert^2$	$\frac{16}{21}\lvert t^{12}_{1\pi^*}\rvert^2$	$\frac{10}{21}\lvert t^{12}_{2\pi^*}\rvert^2$	0
2	±1	∓1	$-\frac{1}{7}\lvert t^{12}_{0\pi^*}\rvert^2$	$-\frac{1}{3}\lvert t^{12}_{1\pi^*}\rvert^2$	$-\frac{10}{21}\lvert t^{12}_{2\pi^*}\rvert^2$	$-\frac{5}{7}\lvert t^{12}_{3\pi^*}\rvert^2$
2	±1	±1	0	$-\frac{2}{7}(t^{12}_{\pm1\pi^*})^2$	0	0
3	0	0	0	$-\frac{1}{6}\lvert t^{12}_{1\pi^*}\rvert^2$	$-\frac{2}{3}\lvert t^{12}_{2\pi^*}\rvert^2$	$-\frac{3}{2}\lvert t^{12}_{3\pi^*}\rvert^2$
3	±1	∓1	$\frac{1}{2}\lvert t^{12}_{0\pi^*}\rvert^2$	$\frac{11}{12}\lvert t^{12}_{1\pi^*}\rvert^2$	$\frac{2}{3}\lvert t^{12}_{2\pi^*}\rvert^2$	$\frac{1}{4}\lvert t^{12}_{3\pi^*}\rvert^2$
3	±1	±1	0	$-\frac{1}{2}(t^{12}_{\pm1\pi^*})^2$	0	0
4	0	0	$\frac{4}{7}\lvert t^{12}_{0\pi^*}\rvert^2$	$\frac{15}{14}\lvert t^{12}_{1\pi^*}\rvert^2$	$\frac{6}{7}\lvert t^{12}_{2\pi^*}\rvert^2$	$\frac{1}{2}\lvert t^{12}_{3\pi^*}\rvert^2$
4	±1	∓1	$-\frac{5}{14}\lvert t^{12}_{0\pi^*}\rvert^2$	$-\frac{3}{4}\lvert t^{12}_{1\pi^*}\rvert^2$	$-\frac{6}{7}\lvert t^{12}_{2\pi^*}\rvert^2$	$-\frac{29}{28}\lvert t^{12}_{3\pi^*}\rvert^2$
4	±1	±1	0	$-\frac{3}{14}(t^{12}_{\pm1\pi^*})^2$	0	0
